# Exploring differences in symptomatic adverse events assessment between nurses and physicians in the clinical trial setting

**DOI:** 10.1038/s41598-023-32123-4

**Published:** 2023-03-25

**Authors:** Lei Liu, Zhanlun Liu, Cuicui Ma, Meng Cheng, Yanli Xie, Lina Zhang, Jianxin Wang

**Affiliations:** 1 Breast Center, Hebei Medical University Fourth Affiliated Hospital, Shijiazhuang, 050011 Hebei Province China; 2 Hebei Province Hospital of Traditional Chinese Medicine, Shijiazhuang, 050011 Hebei Province China

**Keywords:** Breast cancer, Quality of life

## Abstract

A cross-sectional study was performed at Hebei Medical University Fourth Affiliated Hospital from April to July 2020 to explore the difference and consistency between nurses and physicians in terms of symptomatic adverse event (AE) assessment. The Common Terminology Criteria for Adverse Events (CTCAE) was utilized by nurses and physicians to assess patients’ symptomatic AEs. Patients self-reported their AEs utilizing the Patient-Reported Outcomes Version of the Common Terminology Criteria for Adverse Events (PRO-CTCAE). Four nurses and three physicians were enrolled to assess patients’ symptomatic AEs. Given the same AEs, nurses tended to detect more AEs than physicians, and the differences were statistically significant (*P* < 0.001). The toxicity grade reported by nurses and physicians showed no difference for all AEs, except for fatigue (*χ*^*2*^ = 5.083, *P* = 0.024). The agreement between nurses and patients was highest compared to the agreement between nurses versus physicians and physicians versus patients. The differences in symptomatic AE assessment can lead to different symptom management. Thus, it is important to establish a collaborative approach between nurses and physicians to ensure continuity in care delivery.

## Introduction

According to the latest global cancer data, cancer incidence and mortality are increasing rapidly, with an estimated 2.3 million new cancer cases in 2020^1^. Chemotherapy is considered an effective method to stop tumor progression. However, it also has a substantial adverse events (AEs) risk. Accurate and timely reporting of AEs is considered the premise of medical decision-making and has a vital influence on patients’ quality of life. Therefore, AE assessment should be routinely assessed in the clinical setting^2^.

The assessment of subjective AEs has become more structured in recent years. Initially, questionnaires focusing on patient quality of life were used to evaluate subjective adverse events^3^. Currently, many multifaceted adverse event monitoring systems have been established to facilitate the reporting process, such as the direct reporting system of patients adopted by the United States^4^. The system directly incorporates patients' self-reported data into the database for analysis, improving the speed of adverse event collection and providing more robust information for physicians. In the UK, the National Health Service (NHS) implemented patient-reported outcome measures (PROMs) to assess patient outcomes for planned surgical intervention^5^; the American Research Center developed a symptom tracking and reporting system (STAR) to track and report patient adverse events^6^; and the MD Anderson Symptom Inventory (MDASI), developed by the MD Anderson Cancer Center, is the major scale used to collect subjective symptoms of patients in the United States at that time^6^. However, structured AE assessment is still lacking in some countries.

Conventionally, capturing AEs is the responsibility of physicians or nurses utilizing the Common Terminology Criteria for Adverse Events (CTCAE)^7^. Recently, studies have proven the value of incorporating patient self-reported data for symptomatic AE assessment and developed sensitive tools for patients to report their AEs^8–14^. Moreover, information about symptomatic AEs or decision-making is based on reports from physicians or nurses rather than direct reports from patients^15^. Several studies have examined the difference in AE assessment between physicians and patients^10–12,14,16^. However, few studies have investigated the differences between nurses and physicians. It is important to understand the differences between nurses and physicians because different assessments may lead to different symptom management strategies and treatment decisions.

The present study investigated the differences between nurses and physicians regarding symptomatic AE assessment and highlighted the importance of consistency in the medical team. In this paper, we have revealed that given the same symptomatic AEs, the assessments of nurses and physicians differed. Nurses tended to report more AEs than physicians, and the consistency between nurses and patients was higher than that between nurses versus physicians or physicians versus patients. Therefore, this study suggests a more precise collaborative approach is needed to ensure comprehensive care.

## Results

### General data

Between April and July 2020, 417 breast cancer patients were invited to participate in the study. Of these, seven patients were illiterate and five patients refused to participate, resulting in 405 patients enrolling in the study. During the process of data sorting, 21 pairs of questionnaires that were not completed on the same day were eliminated. The remaining 384 sets of nurse-physician–patient questionnaires were available for analysis. Three physicians (all attending oncologists with a master’s degree) and four nurses (two with a master’s degree and two with a bachelor’s degree) who have obtained the national GCP certificate and the professional qualification certificate of relevant specialties were enrolled in the study.

### Reporting rate of symptomatic AEs

According to whether there was an AE present, all collected data were categorized into two groups: the no AE present group (for AEs with grade 0) and the AE present group (for AEs with grade 1–5). The reporting rate of each symptomatic AE was defined as “the number of AEs with grade 1–5 divided by the number of AEs with grade 0–5”. Table 1 shows the type of toxicity according to the grade reported by nurses, physicians, and patients.

**Table 1:** Grades according to questionnaires completed by nurses, physicians, and patients.

Adverse event	Nurse (CTCAE)			Physician (CTCAE)			Patient (PRO-CTCAE)				
	0	1	2	0	1	2	0	1	2	3	4
Nausea/frequency	104	198	82	188	147	49	58	140	134	44	8
Nausea/severity	104	198	82	188	147	49	73	185	97	19	10
Vomiting/frequency	180	146	58	235	118	31	128	128	105	21	2
Vomiting/severity	180	146	58	235	118	31	144	142	74	18	6
Diarrhea/frequency	196	125	63	327	44	13	160	119	83	19	3
Fatigue/severity	101	186	97	292	72	20	30	180	147	24	3
Fatigue/life interference	101	186	97	292	72	20	53	167	137	21	6
Pain/frequency	155	158	71	243	108	33	82	119	130	45	8
Pain/severity	155	158	71	243	108	33	103	166	89	22	4
Pain/life interference	155	158	71	243	108	33	133	133	92	24	2
Constipation/severity	257	95	32	358	18	8	200	131	38	12	3

**Figure 1:**
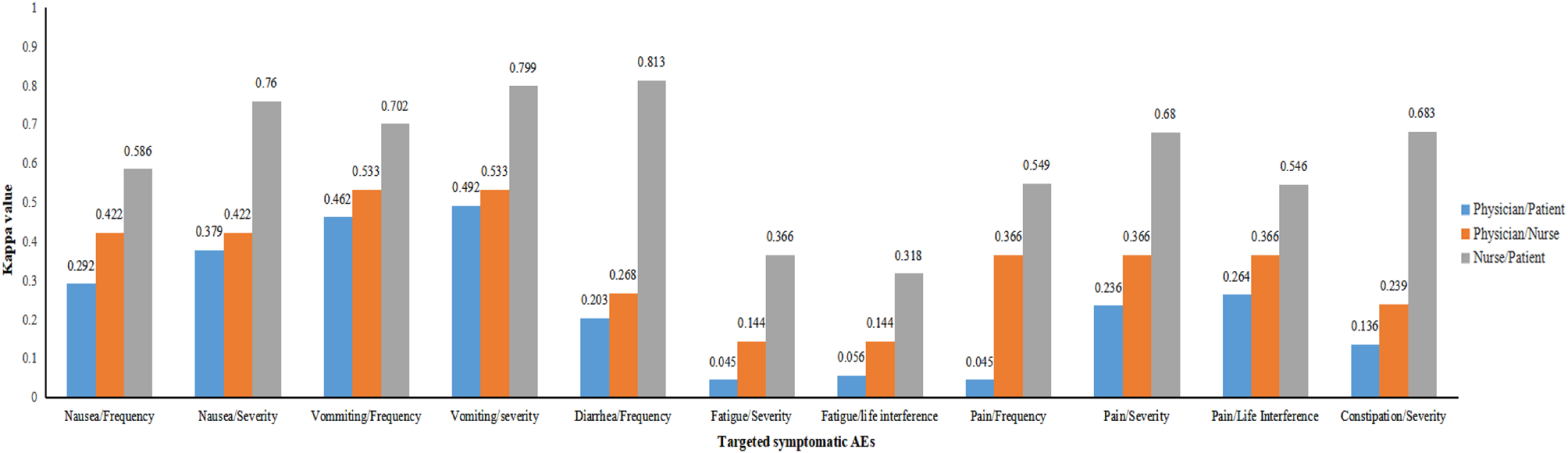
Consistency analysis of symptomatic AEs reporting rate among nurses, physicians, and patients. *k* < 0.000 Poor agreement; 0.000 ≤ *k* ≤ 0.200 Slight agreement; 0.210 ≤ *k* ≤ 0.400 Fair agreement; 0.410 ≤ *k* ≤ 0.600 Moderate agreement; 0.610 ≤ *k* ≤ 0.800 Substantial agreement; 0.810 ≤ *k* ≤ 1.000 Perfect agreement^17^.

A chi-square test was performed to analyze the differences in reporting rates between nurses and physicians. In the analysis, it was noted that nurses reported more AEs than physicians for all targeted symptomatic AEs, and the differences were statistically significant (*P* < 0.001, Table 2). Cohen's kappa coefficient was utilized to analyze the consistency among nurses versus patients, nurses versus physicians, and physicians versus patients. The results showed that the reporting rate for all symptomatic AEs for the nurse versus patient pair was consistently higher than that for the other two pairs (Fig. 1).

**Table 2:** Comparison of reporting rate between nurses and physicians.

	Nausea	Vomiting	Diarrhea	Fatigue	Pain	Constipation
Nurse	72.9%	53.1%	49.0%	73.7%	59.6%	33.3%
Physician	51.0%	38.8%	14.8%	24.0%	36.7%	47.9%
*χ*^2^	38.988	89.280	102.858	190.110	40.387	80.503
*P*	< 0.001	< 0.001	< 0.001	< 0.001	< 0.001	< 0.001

### Toxicity grade of symptomatic AEs

**Figure 2:**
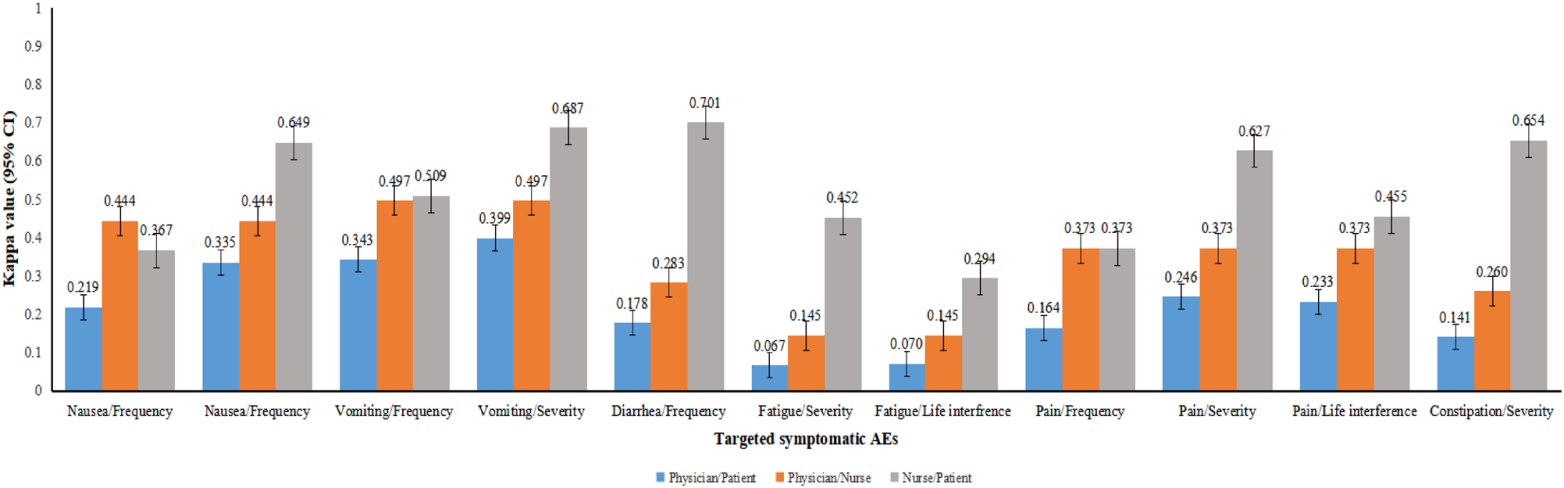
Consistency analysis among nurses, physicians, and patients according to toxicity grade (95% CI).

Table 1 indicates the type of toxicity according to the grade reported by nurses, physicians, and patients. The highest score for nurses and physicians for six symptomatic AEs was 2, whereas the highest score for patients was 4. The Cohen’s kappa coefficient was calculated as a measurement to analyze the agreement between nurses versus patients, nurses versus physicians, and physicians versus patients. The consistency for nurse/patient scored the highest for almost all AEs among the three pairs, except for the frequency of vomiting and pain, which were as consistent as the nurse/physician pair (Fig. 2). We further compared the toxicity reported by nurses and physicians, performing a chi-square test, and found no statistically significant difference in the severity of nausea (*χ*^*2*^ = 1.062, *P* = 0.303), vomiting (*χ*^*2*^ = 2.656, *P* = 0.103), diarrhea (*χ*^*2*^ = 2.342,* P* = 0.126), pain (*χ*^*2*^ = 2.494, *P* = 0.114), and constipation (*χ*^*2*^ = 0.347, *P* = 0.556). However, there was a statistically significant difference in the severity of fatigue (*χ*^*2*^ = 5.083, *P* = 0.024) (See Table 3).

**Table 3:** Comparison of toxicity reported by nurses and physicians.

	Nausea		Vomiting		Diarrhea		Fatigue		Pain		Constipation	
	1	2	1	2	1	2	1	2	1	2	1	2
Nurse	147	49	118	31	44	13	72	20	108	33	18	8
Physician	198	82	146	58	125	63	186	97	158	71	95	32
*χ*^2^	1.062		2.656		2.342		5.083		2.494		0.347	
*P*	0.303		0.103		0.126		0.024		0.114		0.556	

## Discussion

This study explored the differences between nurses and physicians regarding the assessment of symptomatic AEs. The results showed that given the same symptomatic AEs, the assessments of nurses and physicians differed. For the reporting rate of symptomatic AEs, nurses tended to report more AEs than physicians (*P* < 0.001), and the consistency between nurses and patients was higher than that between nurses versus physicians or physicians versus patients. Physicians’ tendency to underreport subjective AEs has been proven by many studies^18–20^. A study found that physician reporting was neither sensitive nor specific in detecting adverse events, even in a tightly controlled clinical trial^20^. The possible reasons for the higher underreporting rate might be the tools used for collecting patient data. Previous studies collected patient data by extracting information from quality-of-life questionnaires. This data extraction and conversion method may lead to misunderstanding and loss of information. Therefore, to avoid information omission, the PRO-CTCAE was used in this study to facilitate an intuitive and sensitive self-assessment. In addition, a previous study found that nurses, physicians and patients had different focuses in adverse event assessments^2^. Physicians tended to focus on adverse events caused by chemotherapy, while nurses and patients tended to report any symptoms, even if they were more likely caused by the disease rather than by the treatment itself. However, we believed it was difficult to distinguish which symptoms were solely caused by treatments and which were caused by disease in the current study. Different educational backgrounds might be a reason for the higher consistency between nurses and patients. The American Nurses Association (ANA)^21^ emphasizes that nurses have mastered assessment skills through professional knowledge. Studies have shown that nurses have the ability to collect data from patients’ perspectives and advocate for their rights^22,23^. Nurses view themselves as patient advocates, and patient safety takes precedence over all other interests^24^. In contrast, physicians focus on therapeutic effects^2^ and treatment-related AEs to identify a more effective treatment approach. It is true that only measuring AEs caused by treatments is not sufficient. Symptomatic AEs are very important and cannot be ignored, as they are mostly the emotional expression of patients’ cognitions of themselves and their disease. Patients’ levels of satisfaction can be measured with therapy by evaluating any improvements in patients’ qualities of life and perceptions of health^25–28^.

Another alternative explanation for the higher consistency between nurses and patients could be nurses’ better communication skills. A study found that some patients are reluctant to report AEs since the more severe their AEs are, the higher the chance of drug dosage adjustment^12^, which in turn may affect the therapeutic effect^29^. In the present study, the underreporting rate among physicians ranged from 49 and 93.2%, while physicians assessed patients’ AEs based on patients’ descriptions. Therefore, we believe that patients’ words may have some impact on the physician’s judgment. In the real world, physicians usually assess patients’ AEs during a medical visit, which lasts approximately 10 min; this limited window is insufficient for patients to fully convey their issues. In the present study, patients were aware of the symptomatic AEs that they needed to discuss with the medical team, and a possible bias may arise because the rate of AEs reported by physicians may be much lower in the real world. Furthermore, nurses have a unique position in a clinical setting to monitor patients’ discomfort. For instance, medication care is part of a nurse’s daily work. Nurses need to closely observe and report all reactions of patients, even if they are more likely due to disease than due to antitumour treatment. Therefore, nurses can be considered the first health care professionals to work with patients when they experience discomfort. Nurses are also often the first health care professionals to work with research patients on a new intervention, drug, or device^30^.

The Kappa value decreased for all pairs when the toxicity grade was considered. The highest scores for nurses, physicians, and patients were two, two, and four, respectively. Medical decisions are made when serious AEs with grades 3 or higher are reported. In this study, there were no changes in medical decisions due to severe AEs. Our findings are consistent with the study performed by Cirillo et al.^2^. Cirillo et al. showed that the lack of measurement of patients’ quality of life could lead medical staff to underestimate adverse events. Another possible explanation for the grade difference could be the different assessment tools utilized. The CTCAE specifies the frequency of AEs that occurred, and each corresponding treatment measure was taken according to the scoring level. Therefore, only when AEs meet the CTCAE criterion can they be graded as 3 or higher, whereas the PRO-CTCAE focuses on patients’ subjective perceptions of health and quality of life. However, we believe that utilizing different assessment tools is inevitable due to different educational backgrounds between the medical team and the patient. In the present study, the most sensitive tools for the medical team and patients were utilized to attempt to reduce this impact, and the PRO-CTCAE was developed from the same content as the CTCAE. On this basis, the grade differences between nurses and physicians were further compared. There were no significant differences between nurses and physicians for most of the AEs except for fatigue, which may be attributed to different reporting rates for nurses and physicians (73.7% vs. 24.0%). Moreover, the grade difference between patients and the medical team revealed the indispensable roles of patients, nurses, and physicians. Patients are the source of their symptomatic AEs, whereas decision-making still depends on the assessment of nurses and physicians. Therefore, a collaborative approach was needed to ensure that comprehensive care was delivered.

This study does have some limitations. First, the time was not recorded for nurses and physicians when they assessed the patients because the study was designed to stimulate clinical practice in the real world. This could result in a bias of the results. Future studies should evaluate the effects of time. Second, the results of this study represent a relatively small sample size from a single centre consisting of a single disease-type patient with relatively good performance status, and the medical team only enrolled four nurses and three physicians to assess patients’ symptomatic AEs; therefore, the generalizability of the study is limited. A multicentre study with more enrolled participants should be conducted to further confirm the results of this study.

## Conclusions

The results of our study suggest the differences between nurses and physicians regarding symptomatic AE assessment and point to the importance of consistency in the medical team. Future studies should be conducted to explore the best ways to integrate those rating resources to ensure consistency and provide vital information required for medical decision-making.

## Methods and materials

### Participants and method

A single-centre questionnaire-based study was conducted in the day chemotherapy ward of the breast centre at Hebei Medical University Fourth Affiliated Hospital from April 2020 to July 2020. Breast cancer patients who had undergone chemotherapy were enrolled in this study, whereas patients who were illiterate or had hearing or visual impairments were excluded. Nurses and physicians with good clinical practice (GCP) were asked to complete online training on adverse event evaluation organized by the Department of Clinical Pharmacology at Hebei Medical University Fourth Affiliated Hospital within 2 weeks. The study only enrolled nurses and physicians who completed the course and passed the test for AE assessment.

To directly collect symptomatic adverse events from patients, we developed a questionnaire based on the PRO-CTCAE. Every questionnaire included six targeted AEs, and patients needed to self-report targeted symptomatic AEs through a website system before the round of chemotherapy started. Medical staff provided no specific assistance when patients completed their questionnaires, although they were always available to clarify questions. The general information, reporting rate, and toxicity grade of all targeted symptomatic AEs for the past 7 days were obtained from all enrolled patients.

Following self-reporting by patients, a paper case report form prepopulated with the same targeted AEs was used for nurses and physicians to assess every patient’s discomfort. All enrolled patients were interviewed during medical rounds separately by nurses and physicians, and the toxicity was graded according to CTCAE version 5.0^31^. Every enrolled patient was requested to describe every symptomatic AE for the past seven days when assessed by nurses and physicians. Therefore, there were three sets of questionnaires: nurses’, physicians’, and patients’ questionnaires, and all questionnaires were collected immediately by nurses once completed. All three parties were not allowed to access each other’s answers. We set every nurse–patient–physician paired questionnaire as a number for the statistical analysis. Reports from other professionals, such as pharmacists, were excluded from the analysis.

### Investigating tools

Patient-Reported Outcomes Version of the Common Terminology Criteria for Adverse Events (PRO-CTCAE) was created as a companion to CTCAE by NCI by extracting all symptomatic AEs from CTCAE. It comprises 124 items representing 78 symptomatic symptoms. In the PRO-CTCAE, there are one to three items for each symptom^8,32^: nausea (frequency, severity), vomiting (frequency, severity), diarrhea (frequency), fatigue (severity, interference with daily lives), pain (frequency, severity, interference with daily lives), and constipation (severity). PRO-CTCAE is more patient-centered and focused on the impacts on patients’ emotions and function. In PRO-CTCAE, all subjective AEs were scored according to attributes: frequency (never = 0, rarely = 1, occasionally = 2, often = 3, almost constantly = 4); severity (none = 0, mild = 1, moderate = 2, severe = 3, very severe = 4); and life interference (not at all = 0, a little bit = 1, somewhat = 2, quite a lot = 3, very much = 4).

CTCAE was developed by the National Cancer Institute (NCI). It classifies all AEs into three categories: AEs based on laboratory results (e.g., hemolysis or neutropenia); measurable/observable AEs (e.g., hearing impairment or retinal tear); and symptomatic AEs (e.g., nausea or fatigue)^4^. Each AE is graded from 1 to 5 to assess the severity of AEs. In CTCAE, the physicians and nurses rated the symptomatic AEs of patients on a five-point scale: score 0 = absent of AEs; score 1 = mild; score 2 = moderate; score 3 = severe; score 4 = life-threatening or disabling; Grading of symptoms in the CTCAE is based on consideration of multiple attributes, including the frequency, severity, and/or interference with activities related to each AE, therefore, one score in CTCAE represents different attributes.

### Targeted symptomatic AEs selection

It is not feasible to assess all symptomatic AEs listed in the PRO-CTCAE, as it may increase patient burden to complete the questionnaire, which, in turn, may affect the quality of the study. Moreover, the results of the study will also be affected by assessing AEs with a low incidence rate. Therefore, a pilot study was conducted first to explore the most common subjective adverse events in breast cancer patients receiving chemotherapy. Of 23 subjective adverse events, nausea, vomiting, diarrhoea, pain, constipation, and fatigue were finally selected^16^. The six targeted adverse events were then transcribed unchanged from the PRO-CTCAE version 1.0 to form a questionnaire (see Table S1). Patients needed to complete the questionnaire through a web-based system to self-report whether targeted adverse events appeared. Moreover, to control the bias of the study, each patient was requested to be assessed by the nurses and physicians at different times on the same day, and the integrity of all questionnaires was checked at the time of completion in case any items were missing. This study was conducted with the approval of the ethics committee of Hebei Medical University Fourth Affiliated Hospital, and written informed consent was obtained from all participants. The study was performed in accordance with relevant guidelines and regulations.

### Statistical method

Statistical analysis was performed using SPSS 25.0 software (SPSSInc., Chicago, IL). The count data were expressed as mean and standard deviation or medians and quartiles. The measurement data were expressed as frequency and percentage. The Chi-square test and Cohen's kappa coefficient were performed to compare the differences and the consistency among three pairs. As the CTCAE and PRO-CTCAE questionnaires have different numbers of items and response options, reporting rate and toxicity grade were analyzed in accordance with their frequency, severity, and impact on the patient’s daily life. When consistency was analyzed between nurses, physicians, and patients, all response options were matched into identical pairs: CTCAE Grade 0 vs PRO-CTCAE Grade 0; CTCAE Grade 1 vs PRO-CTCAE Grade 1; CTCAE Grade 2 vs PRO-CTCAE Grade 2; For pain, nausea, fatigue: CTCAE Grade 3 vs PRO-CTCAE Grade 3 and Grade 4; For constipation, diarrhea, vomiting: CTCAE Grade 3 vs PRO-CTCAE Grade 3, PRO-CTCAE Grade 4 vs CTCAE Grade 4 and Grade 5 combined (See Table S1 for a linear display of possible corresponding questions)^33,34^.

## Supplementary Information

Supplementary Table S1.

## Data Availability

The datasets used and/or analyzed during the current study are available from the corresponding author on reasonable request.
