# Supplementary material for: Exploring differences in symptomatic adverse events assessment between nurses and physicians in the clinical trial setting
Source: Sci Rep. 2023 Mar 25;13:4917. doi: 10.1038/s41598-023-32123-4 (PMC10039895; doi:10.1038/s41598-023-32123-4)
Supplement: Supplementary file 1 — Supplementary Table S1. [file 41598_2023_32123_MOESM1_ESM.doc]

| Targeted symptomatic AEs | CTCAE descriptions | PRO-CTCAE questions |
| --- | --- | --- |
| Nausea | A disorder characterized by a queasy sensation and/or the urge to vomit. | How often did you have nausea? (Frequency) |
|  | A disorder characterized by a queasy sensation and/or the urge to vomit. | What was the severity of your nausea at its worst? (Severity) |
| Vomiting | A disorder characterized by the reflexive act of ejecting the contents of the stomach through the mouth. | How often did you have vomiting? (Frequency) |
|  | A disorder characterized by the reflexive act of ejecting the contents of the stomach through the mouth. | What was the severity of your vomiting at its worst? (Severity) |
| Diarrhea | A disorder characterized by an increase in frequency and/or loose or watery bowel movements. | How often did you have loose or watery stools? (Frequency) |
| Fatigue | A disorder characterized by a state of generalized weakness with a pronounced inability to summon sufficient energy to accomplish daily activities. | What was the severity of your fatigue, tiredness, or lack of energy at its worst? (Severity) |
|  | A disorder characterized by a state of generalized weakness with a pronounced inability to summon sufficient energy to accomplish daily activities. | How much did fatigue, tiredness, or lack of energy inference with your usual or daily activities? (Inference with life) |
| Pain | A disorder characterized by the sensation of marked discomfort, distress or agony. | How often did you have pain? (Frequency) |
|  | A disorder characterized by the sensation of marked discomfort, distress or agony. | What was the severity of your pain at its worst? (Severity) |
|  | A disorder characterized by the sensation of marked discomfort, distress or agony. | How much did pain inference with your usual or daily activities? (Inference with life) |
| Constipation | A disorder characterized by irregular and infrequent or difficult evacuation of the bowels. | What was the severity of your constipation at its worst? (Severity) |

**Table S1.** Linear display for possible corresponding questions for CTCAE and PRO-CTCAE. One scale in CTCAE represents one to three aspects which were matched to PRO-CTCAE items for comparisons.
